# Impact of malnutrition and nutritional support on infectious complications in critically ill infants and children

**DOI:** 10.3389/fped.2026.1913382

**Published:** 2026-10-16

**Authors:** Jason S. Kerstein, Julia K. Weld, Daniel T. Mulhall, Jennifer J. Lee, Anastasia E. Allfather, Sarah U. Morton, Enid E. Martinez, Kimberly I. Mills

**Affiliations:** 1 Division of Cardiovascular Critical Care, Department of Cardiology, Boston Children’s Hospital, Boston, MA, United States; 2 Department of Newborn Medicine, Boston Children’s Hospital, Boston, MA, United States; 3 Department of Anesthesiology, Critical Care and Pain Medicine, Boston Children’s Hospital, Boston, MA, United States

**Keywords:** congenital heart disease, critical care, infection, neonatology, nutrition, pediatrics

## Abstract

Children with critical illness are vulnerable to nutritional deficits, which may contribute to increased susceptibility to infection, impaired immune function and delayed recovery. Optimizing nutritional support during acute illness is therefore essential to improving clinical outcomes. The complex interplay between critical illness, nutrition and infection is nuanced and requires careful consideration to minimize morbidity and mortality. In this narrative review, we synthesize current evidence and considerations regarding optimizing nutritional management to minimize infectious complications across neonatal and pediatric intensive care settings.

## Introduction

Optimal nutrition therapy is a fundamental component of care for critically ill infants and children. However, patients across the pediatric age spectrum frequently face significant barriers to meeting their nutritional needs. Gastrointestinal pathology, such as necrotizing enterocolitis (NEC), post-chemotherapy enteropathy or ischemia, and the need for diagnostic or therapeutic procedures often necessitate interruptions in enteral nutrition (EN). While relying on parenteral nutrition (PN) can provide essential energy and protein requirements, it is associated with complications including bloodstream infection (BSI), fluid overload and gastrointestinal dysfunction. EN is often preferred as it supports intestinal integrity and immune function; however, altered physiologic feeding patterns in the critical care environment are common. Together, critical illness and nutrition-related interventions can lead to gut dysbiosis, endothelial barrier dysfunction, inflammation and increased susceptibility to infection. Conversely, infection and inflammation can further impair nutritional status, creating a complex bidirectional relationship between nutrition and infectious morbidity. In this narrative review, we utilize our clinical and research expertise across the NICU, PICU, and pediatric CICU together with key literature, predominantly from the last 10–15 years together with seminal earlier works, to examine the interplay between nutrition and infection in critically ill infants and children and discuss strategies to optimize clinical outcomes.

### The microbiome in pediatric critical illness

The gastrointestinal tract plays a central role not only in nutrient digestion and absorption, but also in immune regulation, metabolic homeostasis and bidirectional communication with distant organs, including the central nervous system (1, 2). Gastrointestinal health depends on the integrity of the epithelial barrier, coordinated entero-endocrine and neuro-immune signaling, a diverse microbiome, and a responsive immune system. Together, these interconnected systems regulate nutrient utilization, immune development and host defense (1, 3–5).

Critical illness disrupts gastrointestinal homeostasis through inflammation, periods of fasting, and exposure to medications such as sedatives, opioids and antibiotics. Disease-specific factors, including intestinal immaturity in premature infants or impaired mesenteric perfusion in congenital heart disease (CHD), further contribute to epithelial barrier dysfunction (4, 6), altered neuroendocrine signaling (7), dysbiosis (8), and immune dysregulation. These changes can increase susceptibility to infection. Loss of epithelial integrity and dysbiosis may facilitate pathogen overgrowth and bacterial translocation, while altered immune and neuroendocrine signaling can promote systemic inflammation and impair host defenses (7, 9).

Nutrition plays a critical role in maintaining and restoring gastrointestinal function during critical illness. Compared with fasting, EN supports epithelial integrity, immune function and a diverse gut microbiome (10). Nutrient composition and mode of administration may also influence diversity and health of microbial communities with downstream inflammatory pathway impact. Conventional infant and pediatric formulas are often low in fiber and contain predominantly bovine-derived proteins, which may reduce the abundance of short-chain fatty acid-producing commensal bacteria and contribute to a pro- inflammatory intestinal environment (11). Although, physiologic feeding strategies, such as bolus feeding, may better support enteroendocrine and metabolic signaling, the optimal nutritional approach to preserve gastrointestinal homeostasis during critical illness remains uncertain (12).

The relationship between the microbiome and clinical outcomes has been well studied in children with CHD. Alterations in microbiome composition, inflammatory pathways, and epithelial function have been identified before and after cardiac surgery (13–17). Children with CHD exhibit reduced microbial diversity, increased abundance of pro-inflammatory bacteria, and disruptions in immune signaling, with additional effects from medication exposure and physiologic perturbations (14–16). These microbiome alterations have been associated with clinically important outcomes, including feeding intolerance, NEC and postoperative complications (18–21). Collectively, these findings highlight the central role of gastrointestinal health in critical illness and suggest that nutritional strategies targeting the microbiome may represent an important avenue to reduce infectious and inflammatory complications.

## Pediatric intensive care unit

Children present to the pediatric intensive care unit (PICU) with a wide range of medical and surgical conditions, and infection may be either the primary reason for admission or a secondary complication of critical illness. Nutritional considerations therefore vary according to both the underlying disease process and the risk of infectious complications. In this section, we focus on pediatric sepsis and hematopoietic stem cell transplant (HSCT), two populations at particularly high risk for infection and nutrition-related morbidity.

### Pediatric sepsis

Sepsis is characterized by a dysregulated host response to infection resulting in systemic inflammation, hemodynamic instability and multiorgan dysfunction. Although pediatric sepsis affects approximately 1.3% of children in the United States, it remains associated with substantial morbidity and an in-hospital mortality rate of approximately 10% (22). Gastrointestinal dysfunction is common during sepsis and results from a combination of systemic inflammation, bowel wall edema, impaired perfusion, antibiotic exposure, fasting and vasopressor therapy. These factors can contribute to dysbiosis, impaired epithelial barrier function and enteral feeding intolerance, potentially exacerbating systemic inflammation and organ dysfunction (23).

Early EN has been proposed as a strategy to preserve gastrointestinal integrity and improve clinical outcomes in pediatric sepsis. While adult studies have demonstrated mixed results regarding the impact of EN on clinical outcomes in sepsis, pediatric data suggest a potential benefit. In a single-center retrospective study of children admitted to the PICU, both early (</=48 h from admission) and late (>48 h from admission) initiation of EN were associated with improved survival, more ventilator-free days and shorter intensive care unit (ICU) length of stay when compared to no EN after adjustment for severity of illness (24). Concerns regarding bowel ischemia frequently delay initiation of EN in patients receiving vasopressors with sepsis; however, available retrospective pediatric studies have not demonstrated increased harm (25, 26).

While literature regarding sepsis specifically is somewhat limited, guidelines exist for critically ill children in general. American Society for Parenteral and Enteral Nutrition (ASPEN) guidelines recommend EN initiation ideally within the first 48 h of PICU admission with the goal of at least two-thirds of prescribed daily energy requirement by one week into ICU admission, ideally measured through indirect calorimetry. They also recommend a minimum of 1.5 g/kg/day of protein, though there is substantial variability by age in terms of the needs to maintain a positive nitrogen balance (27, 28). Current European Society of Pediatric and Neonatal Intensive Care (ESPNIC) guidelines recommend early enteral nutrition across pediatric age groups in patients receiving hemodynamic support, including vasoactive medications, when hemodynamic status is adequately supported and stable (29). Multiple studies note that despite these goals, rates of adequacy remain low (30, 31). Lower nutritional adequacy has been associated with increased mortality in pediatric studies, although associations differ between protein and energy delivery (30–33). Specifically, inadequate protein intake has been associated with increased 60-day mortality, while results regarding energy adequacy have been more variable (31–33). Early EN has demonstrated decreased mortality in patients with acute respiratory distress syndrome (32) and adequate energy and protein intake by day seven has been associated with decreased 60-day mortality (33). In terms of considering PN, the PEPaNIC trial revealed that delaying initiation of supplemental PN until day eight, compared with initiation within the first 24 h, was associated with decreased risks of infection and ICU length of stay (34). Taken together, these data are reflected in the Surviving Sepsis Campaign guidelines, which recommend initiating EN following adequate hemodynamic resuscitation when vasoactive requirements are stable or decreasing, with gastric feeding preferred, and suggest withholding PN for up to seven days when appropriate. Notably, the guidelines could not determine whether trophic feeding followed by gradual advancement is preferable to early full EN (35). Additional prospective studies are needed to define the optimal timing, composition and delivery of nutrition in pediatric sepsis.

Immunonutrition has been considered as an approach to modulate the inflammatory response in critical illness. Studies have considered targeted amino acids such as glutamine and arginine, vitamins such as Vitamin D and the balance between omega-6 and omega-3 fats. A summary on the mechanisms by which these immunonutrients may be beneficial is beyond the scope of this review. Evidence is very limited in pediatric critical illness, with few randomized controlled trials, which have generally not found a benefit (36–42). Importantly, some large adult randomized controlled trials have found harm with immunonutrients including glutamine and arginine (43–45). The American Society for Parenteral and Enteral Nutrition/ Society of Critical Care Medicine (ASPEN/SCCM) and ESPNIC nutrition guidelines do not recommend the use of immunonutrients at this time (27, 29). Challenges surrounding immunonutrition include the inability to reliably determine levels of many immunonutrients during critical illness due to intracellular flux, fluid overload and changes in protein levels including albumin, which is critical for the transport of some immunonutrients.

### Hematopoietic stem cell transplant (HSCT)

Children undergoing HSCT are particularly vulnerable to both malnutrition and infectious complications. Approximately 15%–25% require intensive care admission, often due to sepsis, respiratory failure or multiorgan dysfunction, with an average length of stay ranging between 3 and 16 days depending on patient outcome (46, 47). The prevalence of comorbidities for patients undergoing HSCT has increased over time and gastrointestinal related comorbidities account for nearly one third of all comorbidities (46). Similarly, the rate of development of multiorgan dysfunction syndrome (MODS) has also increased over time (46). These represent risk factors for nutrition difficulties, particularly EN intolerance. Conditioning with chemotherapeutic agents with or without whole body irradiation causes gastrointestinal epithelial injury manifesting as epithelial barrier disruption, and mucositis and poor enteral intake (48). Conditioning in addition to poor enteral intake and exposure to antibiotics, whether prophylactically or in the setting of fever, also contribute to dysbiosis (49). Epithelial barrier disruption and dysbiosis in an immunocompromised patient substantially increases their risk for infection, particularly bacteremia and sepsis (50, 51). EN, however, has the potential to modify these pathological changes to the gastrointestinal tract. EN, whether administered orally or via a feeding tube, include key prebiotics (e.g., fiber) or even supplemental postbiotics can help recovery of the microbiome and therefore the gastrointestinal epithelial barrier (48, 49). In a single-center retrospective study of pediatric patients who underwent HSCT, those who received greater than 7 days of EN vs. those who received PN alone or PN with less than 7 days of EN had a lower incidence of bloodstream infections despite a similar duration of antibiotics. This study shows the potential role of EN in minimizing the degree of dysbiosis and supports gastrointestinal homeostasis (52).

## Neonatal intensive care unit

Neonates, especially those that are preterm, represent a unique population in terms of their nutritional needs and risks for infection. Necrotizing enterocolitis (NEC) is a known complication in premature infants. In addition to a discussion of neonatal NEC, we discuss neonatal sepsis and the nutrition implications and considerations in this population.

### Nutrition and necrotizing enterocolitis

NEC is a devastating gastrointestinal emergency that affects approximately 5%–10% of very low birthweight (VLBW) infants (<1,500 g), with even higher rates among infants born before 28 weeks' gestation (53). There is a mortality risk of 20%–30% for medical NEC (not requiring surgical intervention), and up to 50% for surgical NEC (54). The pathogenesis of NEC is multifactorial, with primary risk factors including gut immaturity and microbial dysbiosis (Figure 1). Weak mucosal immunity and barrier dysfunction via reduced expression of tight junction proteins render the preterm intestine more vulnerable to luminal pathogens (55, 56). In addition, preterm intestinal epithelium is characterized by an exaggerated innate immune response, wherein the preterm epithelium displays a heightened pro-inflammatory Toll-like receptor (TLR4) signaling (56, 57). Altogether, these factors increase the likelihood of pathologic inflammatory responses to bacterial ligands leading to intestinal injury (57). This vulnerability is further compounded by aberrant microbial colonization, which has been shown to consistently precede the onset of NEC. Metagenomic studies have demonstrated reduced microbial diversity and a relative expansion of Proteobacteria (particularly *Klebsiella*, *Enterobacter*, and *Escherichia*) in the days preceding NEC diagnosis, along with depletion of protective anaerobes (such as *Bifidobacterium*) (58, 59).

**Figure 1:**
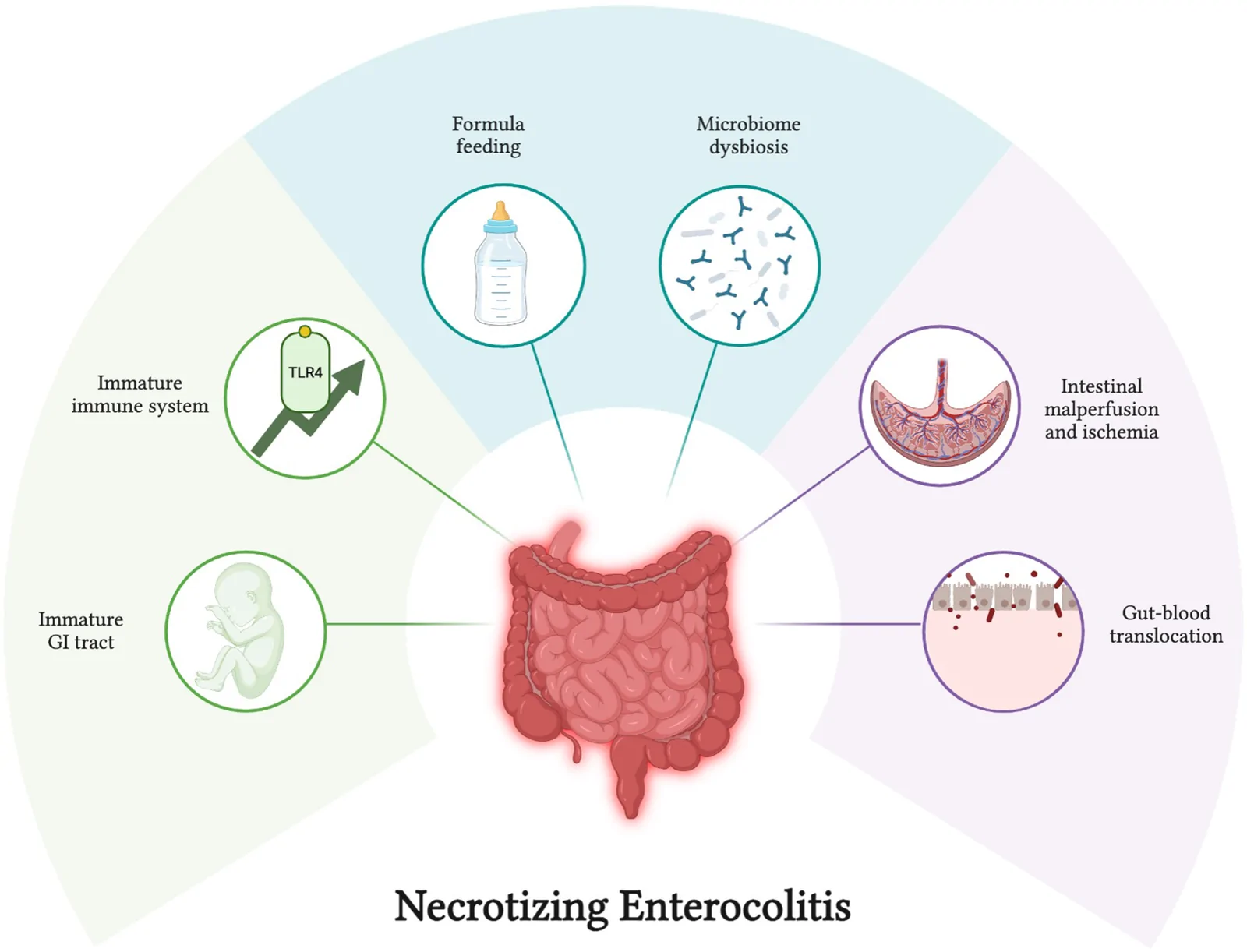
NEC pathogenesis and risk factors.

Both mother's own milk (MOM) and pasteurized donor human milk (PDHM) confer protection against NEC through several complementary mechanisms. For one, human milk contains secretory immunoglobulin A (sIgA), which coats the intestinal epithelium and neutralizes bacterial antigens (60). Lower concentrations of maternal milk and stool IgA have been associated with subsequent NEC development (61). Lactoferrin may further protect against intestinal injury by enhancing gut barrier maturation, sequestering free iron away from bacteria, and exerting direct anti-inflammatory properties (60). Human milk oligosaccharides (HMOs) are also likely beneficial; these selectively promote colonization with beneficial *Bifidobacterium* species (62, 63).

Emerging evidence also highlights the impact of human milk on neonatal immune cell programming. A recent nonrandomized clinical trial using single-cell transcriptomics demonstrated that infants fed human milk had distinct immune cell interleukin signaling profiles from formula-fed infants (64). Specifically, human milk feeding was associated with downregulation of transcriptional signatures linked to allergic inflammation and infection. This suggests that human milk confers systemic immune benefits beyond those seen in the walls of the gastrointestinal tract. In contrast, bovine milk-based formulas can increase intestinal permeability, upregulate oxidative stress signaling, and have direct cytotoxic effects on intestinal epithelial cells (65, 66). A 2020 meta-analysis found that any human milk feeding was associated with a significant reduction in NEC risk as compared to formula (67). This is a dose-dependent relationship; any volume of human milk is better than exclusive formula, and higher doses confer greater protection (68, 69). When MOM is unavailable or limited, PDHM is commonly offered as an alternative. A 2024 Cochrane meta-analysis spanning more than 11 trials and 2,261 infants found that PDHM feeding reduced the risk of NEC by approximately half as compared to formula (relative risk [RR] 0.53, 95% confidence interval [CI] 0.37–0.76) (70). Beyond reducing NEC, any amount of human milk feeding in preterm infants has been associated with reduced rates of late-onset sepsis, retinopathy of prematurity, and improved long-term neurodevelopmental outcomes—with the caveat that these benefits are partly mediated through the reduction of NEC itself (71). Although observational studies and some clinical trials have suggested that an exclusive human milk diet may further reduce the risk of NEC and other complications, the superiority of human milk-derived fortifiers over bovine-based fortifiers remains uncertain, and current evidence is insufficient to support a clear consensus.

### Direct breastfeeding

Robust evidence demonstrates that human milk feeding, including both mother's own milk (MOM) and pasteurized donor human milk (PDHM), reduces infectious morbidity in critically ill infants, including lower rates of late-onset sepsis and necrotizing enterocolitis (NEC) (60, 62, 63, 65, 66, 68, 69). However, studies specifically evaluating direct breastfeeding and infection-related outcomes are limited. Beyond the provision of human milk, direct breastfeeding may confer unique bidirectional immunomodulation between the mother and infant due to retrograde inoculation. In this process, infant saliva is transferred into the nipple and mammary ducts, stimulating tailored immune signaling of maternal antibodies, immune cells and cytokines in response to infant illness. Direct breastfeeding has also been associated with longer duration and greater availability of human milk feeding in term, preterm and congenital heart disease populations (72, 73). Additionally, facilitating direct breastfeeding in critical care settings may reduce maternal physiologic and psychological stress, which may further contribute to improved infant outcomes (74). There is a need to identify interventions to promote direct breastfeeding in the critical care environment with formal guideline development based on future research and quality improvement initiatives.

### Nutrition and neonatal sepsis

Late-onset sepsis, defined as a culture-confirmed bloodstream infection occurring after 72 h of life, affects around 20% of extremely preterm and VLBW infants (75). The gut is now recognized as a leading reservoir for the pathogens commonly causing late-onset sepsis in preterm infants; molecular studies have demonstrated that organisms isolated from blood cultures not infrequently correspond to the dominant organism in the intestinal microbiome at the time of infection (76). These findings support a model of gut-to-blood translocation and implicate intestinal dysbiosis in the development of sepsis, prompting growing interest in microbiome-targeted preventative strategies.

Human milk may reduce the risk of late-onset sepsis through mechanisms that extend beyond its protective effects against NEC. In addition to promoting the growth of beneficial commensal organisms such as Bifidobacterium, human milk contains a broad array of bioactive factors—including secretory immunoglobulin A, lactoferrin, lysozyme, antimicrobial peptides, cytokines, and growth factors—that support mucosal barrier integrity and modulate innate and adaptive immune responses. By fostering a more diverse and resilient intestinal microbiome while simultaneously enhancing host defenses, human milk may reduce bacterial translocation from the gut and decrease susceptibility to systemic infection. Consistent with these biologic mechanisms, observational studies have demonstrated a dose-dependent association between human milk exposure and reduced rates of late-onset sepsis in preterm infants.

Many extremely preterm infants are unable to receive substantial enteral feeds in the first days of life, therefore oropharyngeal administration of colostrum has been investigated as an alternative means of delivering bioactive human milk components. Colostrum exposure to the oral-associated lymphoid tissue may enhance mucosal immune defenses through the actions of secretory immunoglobulins, lactoferrin, cytokines, and growth factors. Although some studies have reported reductions in late-onset sepsis and other adverse outcomes, evidence remains inconclusive, and routine implementation continues to vary across centers.

Probiotic supplementation has emerged as another preventative approach. Multiple randomized trials and meta-analyses have demonstrated that multi-strain probiotic preparations are associated with significant reductions in NEC and all-cause mortality (77). However, implementation of routine probiotic use is complicated by concerns regarding probiotic sepsis (rare but documented, estimated at 8 cases per 20,000 exposed infants (78)), as well as the lack of a U.S. Food and Drug Administration (FDA)-approved, pharmaceutical-grade probiotic product in the United States. The use of strain-specific probiotics in severely immunocompromised patients remains debated and is beyond the scope of this review.

Micronutrient deficiencies may further increase susceptibility to infection. Recent research has focused on zinc, selenium, and vitamin D, all of which are commonly deficient in preterm infants due to interrupted or absent third-trimester placental transfer and increased postnatal demand (79). Zinc is essential for maintaining epithelial barrier integrity, neutrophil and macrophage function, natural killer cell activity, and lymphocyte maturation (80). Selenium serves as a critical cofactor for antioxidant selenoproteins, including glutathione peroxidase and thioredoxin reductase, which protect immune cells from oxidative injury and regulate inflammatory responses (81). Vitamin D influences macrophage and T-lymphocyte activity, with low vitamin D levels being associated with impaired pathogen clearance (82). A recent randomized controlled trial demonstrated that vitamin D, for example, was a useful adjuvant therapy in treatment of term neonates with sepsis (83), but further randomized trials are needed to determine whether targeted supplementation of vitamin D and other micronutrients definitively reduces infection and length of stay (LOS) in preterm infants.

## Pediatric cardiac intensive care unit (CICU)

Neonates up to adults can be admitted to the CICU in the setting of congenital and acquired heart disease. These patients by nature have limited cardiovascular reserve, which greatly impacts both their infectious risk and nutritional needs. Children with CHD are noted to suffer from gut dysbiosis as well as gastrointestinal dysfunction which increases their risk for gut translocation and hospital-acquired infections. In this section, we review some of the unique characteristics of children with congenital and acquired heart disease.

### NEC in children with heart disease

#### Epidemiology

The incidence of NEC in infants and neonates with CHD varies considerably across patient populations and institutions, with reported rates ranging from less than 1% in large heterogeneous CHD cohorts to nearly 20% in high-risk single-ventricle populations undergoing stage I palliation (84–86). Most large studies report an overall incidence of approximately 3%–4% (87–90). The risk of NEC appears to be greatest among premature infants, those with single-ventricle physiology, patients with ductal-dependent heart disease, and those experiencing hemodynamic instability such as low cardiac output or shock (84, 86–91). In contrast, feeding-related practices, including the timing of EN initiation, feeding advancement, and type of EN, have not been consistently associated with the development of NEC (84–87, 90, 91).

When specified in the existing literature, most NEC cases are medical rather than surgical. Although this varies across institution and cardiac lesion, surgical rates are consistently less than 25% of total NEC cases but are associated with substantially higher mortality (87, 89).

Prematurity and circulatory instability are the most consistently identified risk factors for NEC. Large multicenter cohorts, surgical populations, and single-ventricle cohorts alike show increased NEC risk among preterm infants with congenital heart disease (84, 86, 88, 90, 91). Other characteristics associated with NEC include low birth weight, small-for-gestational-age, chromosomal or immunologic abnormalities, and prolonged hypoxemia (85, 86). A recurring finding across the literature is the increased incidence of NEC in single ventricle populations, particularly hypoplastic left heart syndrome (HLHS) (86, 87, 89–91). Similarly, infants undergoing stage I palliation consistently represent a high-risk subgroup, with postoperative NEC rates exceeding those reported in more heterogeneous CHD populations (86, 88, 91).

#### Hemodynamic and ischemic mechanisms of NEC

There continues to be emerging evidence that NEC in patients with CHD is likely a distinct entity from that seen primarily in premature infants. Specifically, NEC in CHD may be in the setting of intestinal ischemia due to low cardiac output, systemic runoff lesions, and regional vasoconstriction. This is supported by data that notes that in infants with NEC, there have been demonstrated differences in the locations affected where infants with CHD were found to have more colonic involvement compared to preterm neonates, thought to be from watershed areas. In this evaluation as well, preterm infants were had lower pH values and higher C-reactive protein values while infants with CHD were of higher postnatal age, again suggesting distinct infectious vs. ischemic etiologies (92). Other studies have demonstrated higher leukocyte and neutrophil counts in infants with CHD and surgical NEC compared to neutropenia in infants without CHD and surgical NEC (93). Surgical specimens in this same cohort demonstrated histology more consistent with an ischemic etiology in infants with CHD as well compared to patients without CHD. Newborns with CHD and NEC demonstrated increased neutrophil elastase and citrullinated histone H3 staining on pathology specimens (93) However, while likely different in etiology, NEC is still currently treated primarily with antibiotics and bowel rest in infants with CHD.

In addition to differences in location and microscopic pathology, patients with CHD have been found to have abnormal blood flow to the gastrointestinal tract. As can be imagined, this is most strongly considered in those with diastolic runoff (for example, in patients with modified Blalock Taussig Thomas (BTT) shunts or ductal stents). But how much of that is related to diastolic steal is somewhat ambiguous. In a cohort of patients who had superior mesenteric artery (SMA) Doppler performed prior to and after a Stage 1, infants with HLHS were noted to have higher resistive indices than normal with no significant difference in preoperative and postoperative diastolic runoff. None of these patients followed developed NEC (94). In contrast, Carlo et al. found that persistent retrograde diastolic flow reversal in the abdominal aorta was associated with NEC. In this case-control study, 47% of cases with NEC had diastolic runoff compared to only 15% of those without and diastolic flow reversal in the abdominal aorta remained the only significant associated factor in multivariate analysis (95).

Infants with CHD have demonstrated endothelial barrier dysfunction, which is why even though the etiology to NEC in cardiac patients may be different it is still treated as primarily infectious. Immunoregulating genes have been demonstrated to be different in infants prior to cardiopulmonary bypass (CPB) and differ after congenital cardiac surgery (13). Compared to control surgery, cardiopulmonary bypass (CPB) has been associated with increases in FABP2 and claudin-3 levels, with decreases in citrulline levels, indicating barrier dysfunction can be attributable to CPB itself (14). Typpo et al. similarly found that CPB induced changes in FABP, citrulline and claudin as well as in sugar permeability. FABP2 and claudin-3 levels increased after CPB while citrulline levels decreased (18). Elevated FABP have been associated with the development of NEC in infants with congenital heart disease (19, 20). Malagon et al. demonstrated that in 7 patients with HLHS who underwent a Stage 1 procedure, all had evidence of impaired gastrointestinal permeability (96). Similar evidence of gastrointestinal permeability has been replicated up to two weeks after congenital cardiac surgery (97). Thus, the combination of CPB and its effects on both endothelial dysfunction predispose children undergoing congenital cardiac surgery to NEC.

#### Relationship between enteral feeding and NEC

NEC in neonates with CHD does not appear to be related to enteral feeding exposure alone. Rather, it comes from a combination of developmental vulnerabilities such as prematurity or low birth-weight combined with CHD and its physiologic impacts. Within that framework, the available evidence does not support routine avoidance of preoperative feeding as a primary NEC prevention strategy, although the quality and granularity of feeding data remain limited.

Large retrospective cohorts, single-center surgical series, and pooled analyses all show no clear association between NEC and preoperative feeding, even in ductal-dependent populations receiving prostaglandin (84, 87, 90, 98–101). This pattern remains similar when feeding is examined in more detail: studies do not show significant relationships between NEC and timing of feed initiation, rate of advancement, caloric density, or type of EN when these variables were assessed (87, 90).

Importantly, withholding feeds does not appear to eliminate risk. NEC occasionally develops before enteral feeding is initiated, suggesting that EN is not a necessary antecedent event to NEC occurrence (85, 87). Feeding details at time of NEC onset are variable and often do not include desired information such as type of nutrition received (human milk vs. formula), caloric density, volume of feeding, rate of feeding advancement, or route of feeding.

Although it is well established that adequate nutrition plays a critical role in reducing the morbidity and mortality in infants with CHD, there are no randomized control trials that identify the optimal feeding strategy in this population from either a nutrition or NEC prevention standpoint.

Braudis et al. examined the implementation and impact of a feeding algorithm in infants with HLHS after surgical Stage 1 palliation at a single center. They found no increased risk of NEC, decreased PN duration, and faster time to goal enteral calories (102). An additional single-center study in neonates with single-ventricle physiology undergoing a hybrid procedure attempted to determine if post-operative gastrointestinal morbidity and NEC rates could be reduced with the implementation of a standardized feeding algorithm (103). They similarly demonstrated that enteral feeds were achieved faster when utilizing a feeding algorithm and found that NEC incidence decreased by half, but this was not statistically significant. Future research is needed to establish effective perioperative feeding algorithms, including the use of both enteral and PN, to optimally nourish and grow this high-risk population and prevent the development of NEC.

#### Risk factors for NEC beyond feeding

In line with the theory of increased malperfusion predisposing to NEC, elements other than feeding are often evaluated for its association with NEC. One large metaanalysis demonstrated that prematurity and HLHS in infants with CHD were risk factors for the development of NEC. Preoperative feeding and umbilical artery catheter placement were not associated with the development of NEC (100). Other studies have demonstrated associations with lower gestational age (84, 90, 91), single ventricle heart disease (84), specifically HLHS (90) as well as truncus arteriosus and aortopulmonary window (90) and from a surgical standpoint higher risk adjustment for congenital heart surgery (RACHS) category (89). NEC has also been associated with the use of packed red blood cell transfusions in term infants with CHD (104).

Data regarding the effect of weight on the development of NEC in infants with CHD has been variable. Becker found that lower birthweight was associated with NEC (84) while a weight of <2.5 kg and the development of NEC in single ventricle infants was associated with mortality (105). Other studies have found no difference in the rates of NEC of survivors vs. non-survivors in infants <2.5 kg (106) nor in small for gestational age vs. not or weight-for-age z-score (107).

#### Management pathways

Although the pathophysiology of NEC in preterm infants and the pathophysiology of NEC in infants with congenital heart disease is unique, NEC definitions and treatment pathways are primarily targeted towards preterm neonates irrespective of CHD. A single center undertook a quality improvement initiative to develop and implement a diagnostic scoring tool and treatment algorithm for NEC in infants with CHD. The study compared 14 patients pre-cardiac NEC scoring tool implementation and 13 patients post-cardiac NEC scoring tool implementation. Thirty-nine total episodes of NEC were analyzed across the two groups. The patients managed with the NEC scoring tool and treatment algorithm had statistically fewer nothing-by-mouth (NPO) days, had a shorter duration of antibiotic treatment, had fewer days receiving PN, returned to full feeds more quickly, and found no increased risk of progression to surgical NEC (108).

#### Outcomes associated with NEC

Although mortality varies considerably across studies, NEC remains consistently associated with substantial morbidity. Infants who develop NEC frequently experience prolonged hospitalization, longer intensive care unit stays, increased rates of ICU readmission, and, in severe cases, a need for surgical intervention (86, 90, 91). Reported mortality among NEC cases ranges from approximately 19% to 47%, with surgical NEC carrying particularly poor outcomes (84, 87). These findings underscore the continued clinical significance of NEC in the CHD population despite its relatively low overall incidence.

### Chylous effusions

Chylous effusions, including chylothorax and chylous ascites, represent a unique example of acquired disease-related malnutrition in children. Most encountered following congenital heart surgery, chylous effusions result in losing nutrient-rich lymphatic fluid containing triglycerides, proteins, fat-soluble vitamins, electrolytes, immunoglobulins, and lymphocytes. Unlike many other causes of pediatric malnutrition, chylous effusions simultaneously impair nutritional status and immune competence through direct depletion of both metabolic substrates and critical components of host defense. As a result, affected children may be particularly vulnerable to adverse infectious outcomes, delayed recovery, and prolonged hospitalization.

Chylothorax occurs in approximately 2%–5% of children undergoing congenital heart surgery and is more common among neonates, infants, and patients undergoing complex procedures (109, 110). Proposed mechanisms include direct injury to the thoracic duct or accessory lymphatic channels, elevated systemic venous pressures that impair lymphatic drainage, and central venous thrombosis or stenosis resulting in lymphatic congestion and leakage. Risk factors include younger age, lower weight, chromosomal abnormalities, single-ventricle physiology, prolonged CPB time, delayed sternal closure, and postoperative venous obstruction (109).

The clinical significance of chylous effusions extends beyond fluid accumulation. Chyle contains substantial quantities of triglycerides, proteins, fat-soluble vitamins, electrolytes, immunoglobulins, and lymphocytes. Persistent chyle losses can therefore lead to protein-energy malnutrition, hypoalbuminemia, essential fatty acid deficiency, deficiencies of vitamins A, D, E, and K, impaired wound healing, and poor somatic growth (111, 112)., These nutritional consequences are particularly important in infants with CHD who frequently have preexisting growth failure and increased metabolic demands before surgery (112).

In addition to nutritional depletion, chylous effusions may contribute to acquired immune dysfunction. Chyle is rich in T lymphocytes and immunoglobulins, and prolonged drainage can result in lymphopenia, hypogammaglobulinemia, and impaired cell-mediated immunity (113). Concurrent protein losses may further compromise immune function through reductions in immunoglobulin synthesis, complement proteins, and other mediators of host defense. Consequently, children with persistent chylous drainage may experience impaired resistance to bacterial, viral, and fungal pathogens.

These nutritional and immunologic derangements provide a biologically plausible mechanism linking chylous effusions with adverse infectious outcomes. Postoperative chylothorax has been associated with increased rates of nosocomial infection, prolonged mechanical ventilation, longer intensive care unit and hospital stays, and increased healthcare utilization (114, 115). Although direct evidence establishing a causal relationship between chylous effusions and infection remains limited, ongoing losses of lymphocytes, immunoglobulins, and nutritional substrates likely contribute to increased susceptibility to infection and delayed recovery from critical illness.

Nutritional management is therefore a cornerstone of therapy. Treatment strategies focus on reducing lymphatic flow while preserving nutritional status. Dietary approaches include restriction of long-chain triglycerides with medium-chain triglyceride-based formulas, defatted human milk, or low-fat diets in older children (112). Because these interventions may further limit nutrient intake, close monitoring is essential to prevent worsening malnutrition and micronutrient deficiencies. In patients with high-output or refractory chylous effusions, temporary bowel rest and total PN may be required. While intravenous lipid emulsions provide an alternative source of calories and essential fatty acids (112), prolonged dependence on PN introduces additional risks, including central line-associated bloodstream infections, further highlighting the complex relationship between nutrition and infection in this population.

The relationship between malnutrition and infection in children is often bidirectional, with infection contributing to nutritional deterioration and malnutrition impairing host defense. Chylous effusions provide a particularly illustrative example of this interaction. Ongoing losses of protein, essential fatty acids, fat-soluble vitamins, lymphocytes, and immunoglobulins can rapidly produce both nutritional deficiencies and immune dysfunction, thereby increasing susceptibility to healthcare-associated infections while simultaneously impairing recovery from existing infections. This convergence of nutritional and immunologic compromise distinguishes chylothorax from many other postoperative complications and underscores its relevance to understanding the impact of nutritional status on infectious outcomes in critically ill children.

Overall, chylous effusions represent a clinically important intersection between malnutrition and immune dysfunction. Through sustained losses of macronutrients, micronutrients, immunoglobulins, and lymphocytes, chylothorax can induce an acquired state of disease-related malnutrition that may increase susceptibility to infection and impair recovery from critical illness. Recognition of this pathway highlights the importance of early nutritional assessment, aggressive nutritional support, monitoring for micronutrient and immune deficiencies, and multidisciplinary management. Future studies are needed to better define the extent to which nutritional rehabilitation can mitigate infection risk and improve outcomes in children with chylous effusions.

### Stress ulcer prophylaxis

The practice of prescribing stress ulcer prophylaxis (SUP) in critically ill infants and children has largely been extrapolated from adult critical care. SUP is intended to reduce stress-related gastric mucosal injury and subsequent upper gastrointestinal (UGI) bleeding. UGI bleeding has been associated with increased morbidity and mortality in critically ill patients (116). However, clinically significant UGI bleeding is uncommon in critically ill infants and children, raising concerns about the safety and efficacy of routine SUP. Acid suppression with histamine-2 receptor antagonists or proton pump inhibitors artificially increases gastric pH, impairing the stomach's natural antimicrobial barrier and promoting intraluminal pathogenic bacterial overgrowth and gut dysbiosis. Retrograde colonization of the oropharynx with potentially pathogenic organisms, particularly gram-negative bacilli, may increase microaspiration and the risk of ventilator-associated pneumonia (VAP) in mechanically ventilated patients. Disruption of the intestinal microbiome and mucosal barrier may also facilitate bacterial translocation into the systemic circulation, potentially contributing to late-onset bacteremia and sepsis in critically ill neonates and infants. Consistent with these mechanisms, SUP has been associated with an increased risk of VAP and *Clostridioides difficile* infection in adults (117). Similar associations with VAP have been reported in critically ill children (118). Among term and preterm infants, SUP has been linked to late-onset bacteremia, NEC and mortality (119–121). Furthermore, critically ill infants with congenital heart disease have demonstrated no increase in UGI bleeding following implementation of restrictive SUP practices (122). Current evidence suggests that the potential infectious risks of SUP may outweigh its benefits in critically ill infants and children, particularly in populations at low risk for clinically significant UGI bleeding.

### Postoperative infections

Postoperative infections are a leading cause of morbidity and mortality in the pediatric CICU, mainly affecting neonates and infants after complex congenital heart surgery. In a multicenter study of over 22,000 CICU encounters, 2.4% of admissions had healthcare-associated infections (HAIs), with nearly 75% occurring in children under one year old (123). Bloodstream infections are the most common postoperative HAIs, closely linked to younger age, prematurity, delayed sternal closure, extracorporeal membrane oxygenation (ECMO) support, prolonged mechanical ventilation, and greater surgical complexity (123–125). These infections are consistently associated with longer hospitalization, increased healthcare use, and worse outcomes (123, 126, 127). Additionally reported HAIs found by Alten et al. included catheter-associated urinary tract infections (1.5/1,000 catheter days), ventilator associated pneumonia (1.9/1,000 ventilator days) and skin and soft tissue infections (0.81/100 operations) (123).

A delayed start of adequate EN often extends the duration of central line use after pediatric cardiac surgery. PN itself is not always an independent infection risk, but prolonged use raises central line exposure and manipulation. Postoperative bloodstream infections affect more than just the acute illness. They often shape the whole recovery process. Studies have shown that patients with HAIs after congenital heart surgery need longer mechanical ventilation, spend more time in the CICU and hospital, use more resources, and have higher mortality (123, 124, 126, 127). Bloodstream infections can also delay nutritional rehabilitation.

An additional site of concern in CHD patient population is skin and soft tissue infections as well as mediastinitis. Rates are reported variably depending on whether superficial and deeper infections are reported together or not. Surgical site infection has been reported from 2%–5% (128–130) with mediastinitis rates reported in one 15-year single-center study looking at over 7,500 surgeries at 0.2% (131). The development of sternal wound infections has been associated with longer CICU and hospital length of stay (129). In addition to the potential to interrupt nutrition with additional procedures in the setting of either washouts or debridement, nutrition is paramount for wound healing in these patients and requires optimization with ongoing treatment of infection.

Hemodynamic consequences of serious infection interrupt feeding advancement, require extra vascular access procedures, and extend PN and antibiotic needs. This creates a cycle—critical illness, delayed nutrition, long central line use, and infection reinforce each other. To break this cycle, postoperative management should combine infection prevention, careful vascular access, and early enteral nutrition. Daily review of catheter need, strict CLABSI prevention, and advancing enteral feeding when possible are key ways to improve nutrition and outcomes (27, 132).

## Access considerations for nutrition and their infectious risks

Current pediatric critical care nutrition guidelines recommend EN as the preferred mode of nutrition whenever feasible, promoting that EN can be safely administered in critically ill patients, including those receiving vasoactive support (27). However, neonates, infants and children who present requiring intensive care, for infectious reasons or otherwise, may not be able to receive EN during their critical illness. Nutrition may be supplemented with PN during these times. PN is a critical component of critical care, providing essential macro- and micro-nutrients until full enteral feeds can be established (133). Given the high osmolality for optimal parenteral provision, this ideally requires central venous access and with that introduces a new infectious risk. Here we discuss the benefits of central access weighed against its risk of inducing central line-associated blood stream infections (CLABSI)**.**

CLABSIs are a consideration in every intensive care unit. The incidence in the NICU is somewhat expectedly inversely related to birthweight (since enteral feeding advances are generally slower in lower birthweight infants), with a rate of 1.56 CLABSIs per 1,000 central catheter-days for neonates with birthweight ≤1,500 g as compared to 0.72 per 1,000 central catheter-days for neonates >1,500 g (134). Catheter insertion and maintenance bundles, with measures such as strict hand hygiene, maximal barrier precautions at insertion, and routine hub care, have been shown to substantially reduce CLABSI rates across NICU settings (135).

In addition to central access considerations, the composition of PN itself may modulate infectious risk. Soybean oil-based intravenous lipid emulsions, which are rich in omega-6 fatty acids and phytosterols, have been associated with enhanced inflammatory responses and oxidative stress in *in vitro* studies (136, 137). In contrast, newer mixed-oil and fish oil-containing lipid emulsions appear to have more favorable profiles, with potential benefits for liver function and other clinical outcomes (137). Specifically, olive oil-based emulsions offer a more balanced fatty acid profile with proven safety in preterm neonates (138), while fish oil-containing mixed-oil emulsions (e.g., SMOF) provide anti-inflammatory, immunomodulatory omega-3 fatty acids associated with fewer infections in critically ill adults (139). In pediatric patients, mixed-oil emulsion use has been associated with significantly lower catheter-related bloodstream infection rates than soybean oil-based emulsions (140), though a Cochrane review found no statistically significant reduction in sepsis risk with alternative emulsions in preterm infants — underscoring the need for adequately powered prospective trials correlating lipid emulsion choice with bloodstream infection incidence in pediatric and neonatal populations (141). However, the impact of these alternative lipid formulations on infection-related outcomes remains uncertain, and high-quality randomized controlled trials are needed to establish their clinical superiority.

The considerations surrounding the use of PN in the PICU are as multifactorial as the patient population it serves. Patients may rely on PN for a variety of reasons ranging from acute presentations with hemodynamic collapse, elective procedures that impact the gastrointestinal tract, or other acquired states that prevent enteral nutrition such as high vasoactive requirements, mechanical ventilation or inability to tolerate enteral nutrition.

Postoperative cardiac patients may rely on supplemental or exclusive PN in the early postoperative period for a variety of reasons including: delayed initiation of enteral feeds, feeding intolerance, fluid restrictions, hemodynamic instability, and prolonged perioperative fasting. As PN requires reliable venous access and often coexists with vasoactive medications, sedation infusions, blood products, and anticoagulation therapies, vascular access planning is an essential aspect of patient and nutritional management.

Healthcare-associated infections (HAIs), particularly CLABSIs remain a major source of morbidity and mortality for patients in the pediatric CICU. In the largest multicenter analysis to date, Alten et al. evaluated 22,839 CICU encounters across 22 institutions and found that 2.4% were complicated by an HAI, with nearly 73% occurring in children younger than one year of age. CLABSI was the most common HAI, occurring at a rate of 1.1 infections per 1,000 line days (123). Increasingly, vascular access selection is being viewed as a longitudinal component of CHD management rather than a decision made solely during the postoperative period. Preoperative access planning offers an opportunity to minimize interruptions and delays in care throughout all anticipated phases of postoperative recovery, including PN, vasoactive medications, and prolonged antimicrobial treatment. Published guidelines for catheter selection, matching the vascular device to the expected duration and intensity of therapy, have been developed through the miniMAGIC-CHD recommendations. The advantage of this approach is that it considers the totality of care for the patient's lifespan, including future catheterization procedures, possible need for mechanical circulatory support, and the risk of central venous obstruction in children with complex congenital heart disease (142). Similarly, Wagner et al. demonstrated that standardized vascular access assessment prior to line placement can improve catheter selection by including expected treatment duration, infusion characteristics, and patient-specific anatomical considerations (143). Such planning is particularly valuable in neonates with anticipated multiple intervention-based strategies (e.g., single-ventricle patients), where preservation of central venous pathways remains critical for future interventions.

Surgically placed transthoracic intracardiac lines, particularly right atrial catheters, remain a common strategy for postoperative nutritional support because they provide reliable central access without requiring additional vascular cannulation. Historical and contemporary studies have demonstrated remarkably low infection rates with these devices when used in the immediate postoperative period. Gold et al. reported an overall complication rate of only 0.59% over a decade of experience (144). Similarly, Damen and Van der Tweel found no catheter-related bacteremia, endocarditis, or positive tip cultures among 279 transthoracic catheters despite observing increased colonization rates with prolonged catheter duration in conventional central venous catheters (145). More recent investigations have continued to support the safety of intracardiac access. Flori et al. reported positive blood cultures in only 1.5% of patients with transthoracic intracardiac catheters (146), while Stein et al. observed no CLABSIs among 176 transthoracic lines placed in neonates following cardiac surgery (147). Anton-Martin et al. further demonstrated excellent outcomes in a cohort of 692 postoperative cardiac patients with right atrial lines, reporting infection rates of only 0.7%, with all infections successfully treated using antibiotics and none requiring catheter removal (148). Although bleeding following catheter removal is a recognized complication, most events are minor and self-limited, and serious adverse outcomes are uncommon (146, 147). Collectively, these results indicate that intracardiac catheters may provide a safe and durable option for PN administration while reducing the need for additional central venous access immediately postoperatively.

Balancing the competing priorities of ensuring adequate nutritional delivery and preventing bloodstream infection requires a multidisciplinary approach involving intensivists, surgeons, nutritionists, pharmacists, and vascular access specialists. Given the strong association between catheter duration and CLABSI development, daily evaluation of line necessity, dedicated PN lumens, avoidance of unnecessary line manipulations or access, and prompt advancement of enteral nutrition are crucial elements of care. Current pediatric critical care nutrition guidelines stress minimizing interruptions to enteral feeding whenever possible and transitioning away from PN as clinical status permits (27). Future quality improvement initiatives should continue to focus on incorporating nutrition goals with vascular access stewardship, ensuring that children receive adequate postoperative nutrition while minimizing the substantial morbidity and mortality associated with central line-associated bloodstream infections.

## Conclusion

The interplay of pediatric critical illness, infection and nutrition spans all intensive care units in both similar and unique ways. While there are many different reasons for which these neonates, children and adolescents present with care, all require careful consideration of their nutritional status. Infection can be a significant consequence of all of these populations and supporting nutrition to assist in prevention and treatment of infection is critical (Table 1). Further research into the optimal methods of nutritional provision during critical infection are needed to optimize recovery and limit morbidity and mortality.

**Table 1:** Nutritional interventions and infectious risk across pediatric critical care settings.

Clinical Setting	Population/Clinical Scenario	Mechanism Linking Nutrition & Illness	Nutritional Intervention	Key Evidence Cited	Gaps
Pediatric Intensive Care Unit (PICU)	Sepsis	Systemic inflammation, bowel wall edema, hypoperfusion, antibiotic/vasopressor exposure → barrier dysfunction, dysbiosis, feeding intolerance → worsened inflammation/organ dysfunction	Early EN (even ≤48 h) reasonable when hemodynamically feasible; vasopressor support alone is not a contraindication	Single-center retrospective cohort Both early (≤48 h) and late (>48 h) EN associated with ↑survival, more ventilator-free days, shorter PICU stay vs. no EN (24)	High-quality RCTs needed to better define optimal EN timing, composition and delivery.
	∼1.3% of pediatric hospitalizations; ∼10% in-hospital mortality (22)				
	Hematopoietic stem cell transplant (HSCT) recipients 15–25% require ICU admission; GI complications account for ∼1/3 of all comorbidities (46, 47)	Conditioning chemotherapy ± irradiation → barrier injury, dysbiosis, immunosuppression → bacteremia/sepsis risk	Prioritize EN (PO or PG) over PN alone/PN-predominant approaches; especially EN with prebiotic fiber or postbiotics to best support microbiome recovery	Single-center retrospective cohort HSCT patients who received EN >7 days had ↓bloodstream infection incidence vs. PN alone/PN-predominant, despite similar antibiotic duration (52)	
Neonatal Intensive Care Unit (NICU)	Necrotizing enterocolitis (NEC) in the VLBW (<1,500 g) and extremely preterm infant (<28 weeks’ gestation); 5–10% incidence; mortality 20–30% in medical NEC, up to 50% in surgical NEC (53, 54)	Gut immaturity, weak mucosal barrier, exaggerated TLR4-driven inflammation → barrier dysfunction, dysbiosis, inflammation → NEC risk	Mother's own milk (MOM) preferred; pasteurized donor human milk (PDHM) if MOM unavailable/insufficient	Meta-analyses Any human milk ↓NEC vs. formula, dose-dependent (67–69) PDHM ↓NEC risk ∼50% vs. formula (70)	Superiority of human milk-derived vs. bovine-based fortifiers remains unresolved.
	Late-onset sepsis in the VLBW and extremely preterm infant; ∼20% incidence (75)	Gut immaturity, weak mucosal barrier → dysbiosis, gut-to-blood translocation → late-onset sepsis risk	Maximize human milk exposure; oropharyngeal administration of colostrum (OPA) when substantial early EN is not feasible; consider probiotic supplementation	Observational studies: human milk ↓LOS (71) Meta-analysis of observational studies and RCTs: probiotics ↓NEC and ↓all-cause mortality (77)	OPA data inconclusive; implementation varies by center. No FDA-approved, pharmaceutical-grade probiotic product available in the United States; strain-specific RCTs needed.
Cardiac Intensive Care Unit (CICU)	Necrotizing enterocolitis in children with congenital heart disease (CHD); 3–4% across all CHD (87–90); up to ∼20% in single-ventricle physiology (84–86)	Circulatory instability and low cardiac output (especially after stage I palliation), diastolic runoff (especially with modified BTT shunts or ductal stents) → intestinal ischemia, barrier dysfunction, regional vasoconstriction (distinct from preterm NEC)	Standardized perioperative feeding algorithms; no need to routinely withhold preoperative feeding (even in ductal-dependent lesions on prostaglandin)	Large retrospective cohorts, single-center series, and meta-analyses: no association between preoperative feeding and NEC (84, 87, 90, 98–101)	Further research needed to establish optimal and effective perioperative feeding algorithms.
	Post-cardiac surgery chylothorax/chylous effusions 2–5% incidence (109, 110)	Loss of triglycerides, protein, fat-soluble vitamins, lymphocytes, immunoglobulins → malnutrition, acquired immune dysfunction (lymphopenia, hypogammaglobulinemia)	MCT-based formula or defatted human milk; low-fat diet in older children; PN/bowel rest if high-output or refractory	Observational cohorts: chylothorax associated with ↑nosocomial infection, prolonged mechanical ventilation, longer length of stay (114, 115)	Causal link to infection not established. Unclear whether nutritional repletion mitigates infection risk–needs further study.
	Healthcare-associated infections (HAIs) in postoperative cardiac patients ∼2% of CICU admissions; CLABSI most common (123)	Delayed start of adequate EN → prolonged PN/central-line dependence → ↑CLABSI risk, delayed nutritional rehabilitation (self-perpetuating cycle)	Early EN when hemodynamically feasible; CLABSI prevention bundles and daily catheter-necessity review; transthoracic (e.g., right atrial) lines as an option for early postoperative PN access	Multicenter analysis: low infection rates (0.6–1.5%) in patients with transthoracic intracardiac catheters (144–148)	Comparative data on access strategies vs. infection/nutrition outcomes are limited.
	
Cross-cutting (any ICU setting)	PN-dependent patients	As above; prolonged central-line dependence → CLABSI risk	As above; can also consider mixed-oil/fish-oil emulsions (e.g., SMOF)	Meta-analyses/cohort data support bundle efficacy for CLABSI reduction (135) Lipid-emulsion data show biologic plausibility for benefit with more favorable inflammatory/liver profiles (137)	High-quality RCTs needed to determine if lipid-emulsion choice reduces infection across ICU settings.
		Soybean-oil lipid emulsions → ↑omega-6 exposure → pro-inflammatory signaling, altered neutrophil function			
	Mechanically ventilated patients	Acid suppression → ↑gastric pH → dysbiosis (gram-negative overgrowth), gut-to-blood translocation, ventilator-associated pneumonia → extends to NEC/LOS risk in neonates	Restrictive stress ulcer prophylaxis (SUP), only use for high UGI-bleeding risk	Meta-analysis (adult), cohort studies (pediatric): SUP linked to ↑VAP, late-onset bacteremia, NEC, mortality (117–121) RCT in infants with CHD: no ↑UGI bleeding with restrictive SUP (122)	Additional RCTs needed across ICU settings.
Overall	All pediatric critical care settings	Nutrition ↔ microbiome ↔ barrier integrity ↔ immune function ↔ infection (bidirectional)	Early, individualized, multidisciplinary nutrition therapy tailored to setting-specific risk	Evidence base is predominantly retrospective/observational. RCTs remain sparse across all populations reviewed.	

BTT, blalock-Taussig-Thomas; CHD, congenital heart disease; CICU, cardiac Intensive Care Unit; CLABSI, central line-associated bloodstream infection; EN, enteral nutrition; FDA, food and Drug Administration; GI, gastrointestinal; HAI, healthcare-associated infection; HSCT, hematopoietic stem cell transplant; ICU, intensive care unit; LOS, late-onset sepsis; MCT, medium-chain triglyceride; MOM, mother's own milk; NEC, necrotizing enterocolitis; NICU, neonatal Intensive Care Unit; OPA, oropharyngeal administration of colostrum; PDHM, pasteurized donor human milk; PG, per gastric; PICU, pediatric Intensive Care Unit; PN, parenteral nutrition; PO, per os (by mouth); RCT, randomized controlled trial; SMOF, soybean oil; medium-chain triglycerides; olive oil; and fish oil; SUP, stress ulcer prophylaxis; TLR4, toll-like receptor 4; UGI, upper gastrointestinal; VAP, ventilator-associated pneumonia; VLBW, very low birth weight.
